# NestedMICA as an ab initio protein motif discovery tool

**DOI:** 10.1186/1471-2105-9-19

**Published:** 2008-01-14

**Authors:** Mutlu Doğruel, Thomas A Down, Tim JP Hubbard

**Affiliations:** 1 Wellcome Trust Sanger Institute, Hinxton, Cambridge CB10 1HH, UK; 2 Wellcome Trust/Cancer Research UK Gurdon Institute, University of Cambridge, Tennis Court Road, Cambridge CB2 1QN, UK

## Abstract

**Background:**

Discovering overrepresented patterns in amino acid sequences is an important step in protein functional element identification. We adapted and extended NestedMICA, an ab initio motif finder originally developed for finding transcription binding site motifs, to find short protein signals, and compared its performance with another popular protein motif finder, MEME. NestedMICA, an open source protein motif discovery tool written in Java, is driven by a Monte Carlo technique called Nested Sampling. It uses multi-class sequence background models to represent different "uninteresting" parts of sequences that do not contain motifs of interest. In order to assess NestedMICA as a protein motif finder, we have tested it on synthetic datasets produced by spiking instances of known motifs into a randomly selected set of protein sequences. NestedMICA was also tested using a biologically-authentic test set, where we evaluated its performance with respect to varying sequence length.

**Results:**

Generally NestedMICA recovered most of the short (3–9 amino acid long) test protein motifs spiked into a test set of sequences at different frequencies. We showed that it can be used to find multiple motifs at the same time, too. In all the assessment experiments we carried out, its overall motif discovery performance was better than that of MEME.

**Conclusion:**

NestedMICA proved itself to be a robust and sensitive ab initio protein motif finder, even for relatively short motifs that exist in only a small fraction of sequences.

**Availability:**

NestedMICA is available under the Lesser GPL open-source license from:

## Background

Discovering linear sequence motifs common to a set of protein sequences has long been an important problem in biology. It is possible to check if a set of proteins contain a known sequence motif by searching protein motif or domain databases. Databases including Pfam [1], eukaryotic linear motif database (ELM) [2], Prosite [3] and ScanSite [4] contain sequence motifs and domains in the form of regular expressions or profile HMMs. Obviously, one cannot use these resources to discover a novel or unannotated sequence motif that is suspected to be a common feature in a given protein set. While new protein domains such as those contained in Pfam can be defined from alignments of evolutionarily related sequences, the identification of short sequence motifs, potentially shared between proteins that appear evolutionarily unrelated, is much harder.

To tackle this problem, several multiple alignment approaches [5,6] have been proposed. One such tool, Dilimot [7], is a recent protein motif search tool aiming at finding relatively short overrepresented motifs by aligning only sequence regions that are likely to contain a linear motif. It filters out regions including globular domains and coiled-coil regions which are reported or predicted by some other algorithm, before searching for known motifs in several protein databases such as PFAM, and finally uses a pattern search program, TEIRESIAS [8] to find overrepresented matches. TEIRESIAS, software that is not based on database look-up, can list frequently repeating character-based patterns that include gaps, from a given sequence set. Patterns can include one or two "events" separated by wild-card characters, as in "AT..G" [9]. Both Dilimot and TEIRESIAS report results as regular expressions. There are also other algorithms in the non ab initio motif finding category, using evolutionary or structural information, which are specifically designed to predict DNA-binding regions in protein sequences [10-12]. However since the MEME tool was developed [13] and provided a way to carry out ab initio protein motif finding, returning a set of Position Weight Matrices (PWMs) rather than regular expressions, not many multi-purpose sequence-based probabilistic motif finders have been developed, despite there being numerous tools for finding motifs in DNA.

NestedMICA [14] is a probabilistic motif discovery algorithm which uses a new Monte Carlo inference strategy called Nested Sampling [15]. Written in the Java programming language as an open source application, NestedMICA uses Biojava libraries [16]. It has been successfully used for transcription binding site and large-scale promoter motif discovery [17]. In this manuscript, we extend the application of NestedMICA to finding motifs in protein sequences and compared it with the popular program MEME using both biologically-authentic and synthetic test data sets. We chose to compare NestedMICA with MEME, because the output of MEME is motifs in the form of PWMs, making comparison possible. MEME is also an ab initio method and uses probabilistic models like NestedMICA.

To evaluate the performance of the two methods we have performed various spiking tests in which some test motifs generated from protein domain alignments were spiked into a set of protein sequences, as described in the Methods. NestedMICA has also been assessed by testing its ability to find a subcellular localisation motif in datasets known to contain a specific localisation signal.

## Results and Discussion

### Background model

**Figure 1:**
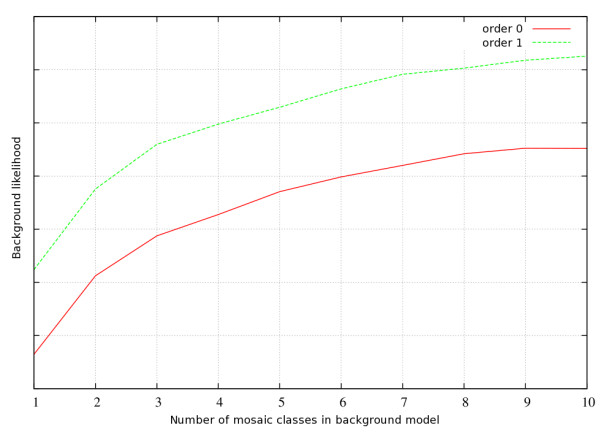
**Likelihood curve for different number of mosaic classes**. The x-axis represents the total number of mosaic classes in the tested background model architecture. The logarithmic y-axis corresponds to a likelihood measure that can take arbitrary values, of how well a background model represents the given sequence set. The red line represents a zero-order while the green one represents a first-order background model.

The first step in using NestedMICA is the generation of a background model to represent the "uninteresting" parts of sequences that do not contain motifs of interest (see Methods). From a series of tests we concluded that different sets of protein sequences vary in complexity and composition too much to develop a generic background model. Most of the time, training a dedicated background model for each protein dataset is the best way to maximise performance and sensitivity. Prior to motif finding, sequence likelihood analysis must be performed to test a variety of background models and select the optimal one. Figure 1 shows one such likelihood curve performed on a set of cytoplasmic proteins. Generally, if there is sufficient data to perform a proper training, using order-1 background models proved to be better than order-0 models for proteins. As far as the number of mosaic classes is concerned, a class number should be picked that falls on the corresponding likelihood curve before it starts to saturate or drop, regardless of whether it increases at a later stage.

### Performance vs. motif abundance

**Figure 2:**
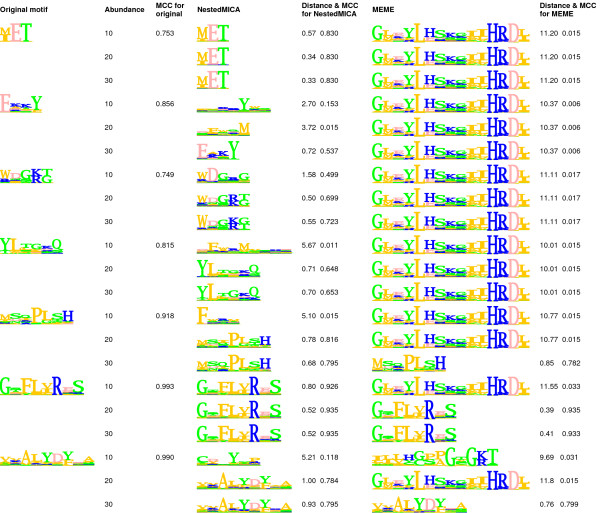
**Motifs recovered by NestedMICA and MEME in the single-motif spiking tests, for motif set 1**. Motifs in this set were obtained from several Pfam domain entries. For each original test motif used in the motif spiking tests, the 3 tested abundance rates are shown in the next column. For motifs recovered by NestedMICA (fourth column) and MEME (sixth column) the cartesian distance to the original test motif and the MCC value obtained when the motif is used for sequence scanning are shown. For comparison purposes, the MCC values of the original test motifs are shown as well. In NestedMICA protein sequence logos, hydrophobic residues are represented in orange, polar and hydrophilic ones in green, acidic ones in pink, and finally basic amino acids are depicted in blue.

We used 3 different motif sets each containing 7 motifs of lengths ranging from 3 to 9 amino acids. Instances of each of the motifs (see Figure 2 for motif set 1, and Additional files 1 and 2 for motif sets 2 and 3, respectively) were separately spiked into the cytoplasmic dataset (see Methods). The 21 motifs were inserted into the sequences at different frequencies (10, 20 and 30%), allowing us to test motif discovery software under different conditions of motif abundance. Generally, performance for both NestedMICA and MEME increased with increasing abundance rate of the inserted motif.

Each of these three figures shows a set of tests performed at different motif abundance rates with the original test motifs, along with the corresponding motifs found by both NestedMICA and MEME. For each motif reported by NestedMICA and MEME, its cartesian distance from the corresponding original motif is given. As Table 1 summarises, low abundance motifs and short motifs were more difficult to recover for MEME, even if they had a high information content. For example, out of the maximum 4.32 bits per position, the average information content per position was 3.96 bits (91.5%) for motif of length 3 in set 2, while it was 3.68 bits (85.2%) for motif of length 4 in the same motif set [see Additional file 1]. Both could not be recovered by MEME at the tested 10, 20 and 30% abundance rates. The motif of length 3, for example, could only be recovered correctly by MEME when it was present in at least 80% of the sequences (data not shown). In contrast, the same motif was recovered by NestedMICA when present in only 10% of the sequences. NestedMICA did not miss any of the 21 motifs when they were present at 30% abundance. It also correctly recovered 95.2% and 61.9% of them when the motif abundance rate was 20%, and 10%, respectively (Table 1).

**Table 1:** Motif recovery performance summary for NestedMICA and MEME. Numbers shown correspond to the correctly recovered number of motifs by NestedMICA (NMICA) and MEME for each test set, each of which contains 7 motifs, for the single-motif spiking tests. Motifs recovered for set 1 can be seen on Figure 2, while the results for the other tests performed using sets 2 and 3 can be found in Additional Files 1, 2. A motif is considered as correctly recovered if the average cartesian distance per residue position between the recovered motif and the original motif that was spiked is < 0.3 (see Methods).

Motif rate (%)	Set 1		Set 2		Set 3		Total correct (%)	
	NMICA	MEME	NMICA	MEME	NMICA	MEME	NMICA	MEME
10	3	0	4	0	6	2	61.9	9.5
20	6	1	7	4	7	3	95.2	38.0
30	7	3	7	5	7	4	100.0	57.1

In addition to cartesian motif distances, measuring the similarity between the recovered motif and the original, the performance of the motifs in finding motif instances when scanning test sequences is indicated by Matthew's Correlation Coefficient (MCC) [18] values (see Figure 2, and Additional files 1, 2). MCC is a single measure that captures performance over a range of sensitivity and specificity values (see Methods). Raw sensitivity and specificity values are given in Additional file 3 for the three motif sets. These measures have been used to evaluate the scanning performances of the original and reported motifs, by testing spiked datasets (independent of the spiked datasets used for training) where each sequence contains an instance of a particular motif. We provide the MCC values for the original test motifs, too, for better interpretation of the MCC values given with the motifs reported by both programs. Having relatively lower sensitivity/specificity values, and hence a lower MCC, does not necessarily mean that a program is not doing well in finding a certain motif, but in certain cases it can indicate that the target motif is a weak one and therefore more difficult to recover. MCC values for the original motifs were calculated in a similar way to the others, i.e., by spiking every sequence in the background test dataset with the generated instances of a particular motif, and then scanning the spiked dataset with the original motif to see how many motif hits would be found using a range of score thresholds (see Methods).

### Performance with multiple motifs

Individual protein sequences may contain multiple different motif of interest. For example, proteins targeted into the endoplasmic reticulum (ER) by an N-terminal Signal Peptide (SP) sequence are maintained in the ER if they have also a " [KH]DEL" retention signal on their C-terminus. After determining the ability of both NestedMICA and MEME to find single motifs, we assessed the two programs' ability to recover multiple motifs from a single dataset.

**Figure 3:**
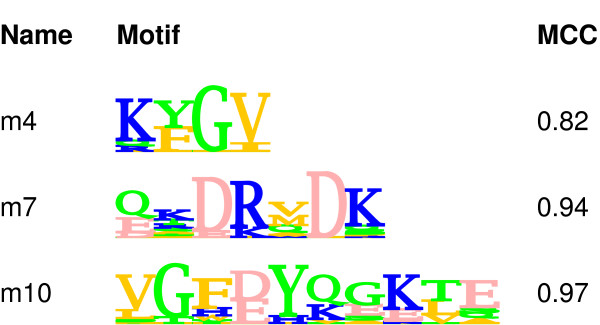
**Inserting more than one different motif into the sequences**. Original motifs used in multiple motif test are shown. These were inserted into the test sequences, at 40 and 20% total motif abundance rates. Resulting spiked sequences contain either zero, one or multiple different instances of the shown motifs, while sequences were not allowed to contain multiple instances of the same motif. The MCC values of these original motifs are given for comparison with the recovered motifs' MCCs. Results for recovered motifs are presented in Table 2.

We used 3 test motifs of length 4, 7 and 10 aa, in the multiple motif spiking tests (Figure 3). Multiple motifs were spiked in such a way as to ensure an unbiased distribution. For example, in the first multiple motif spiking test, corresponding to a 40% abundance rate for each motif, it was ensured that 24% of the sequences were spiked with only motif of length 7, 24% only with motif of length 10 and 16% with both motifs. This corresponds to the distribution of motifs that would be expected by chance. The test was repeated by halving the total abundance rate for each motif.

In a similar way, two other pair combinations of the motifs were tested, and finally, three motifs were spiked at the same time. When the abundance rate for each spiked motif in the triple motif test was 40%, it was ensured that three different groups of sequences, each corresponding to 14.4% of the total, contained either motif of length 4, or 7 or 10; three different groups, each corresponding to 9.6% of the total contained two motif instances simultaneously (i.e. one group had both motifs of length 4 and 7, another had both 7 and 10, and finally another had both 4 and 10) and one group corresponding 6.4% contained all three motifs.

Table 2 summarises the performances of both programs for the multiple motif finding tasks performed under different conditions. It shows the cartesian distances and MCC values of the reported motifs (The corresponding sensitivity and specificity values are given in Additional file 3). In general, both NestedMICA and MEME performed well, except MEME had a tendency not to recover shorter motifs and instead report PWMs of maximum allowed length which did not correspond to any of the spiked motifs.

**Table 2:** Performance summary for the programs in the multiple motif spiking tests. The "distances" columns refer to the cartesian distances between the reported motifs and the original ones which are shown in Figure 3. Motif names indicate length. Total abundance rates (in percent) for the spiked motifs are provided. In addition to cartesian distances, MCC values are given for both programs. For the MCC values of the original test motifs, refer to Figure 3.

Motifs	Rate (%)	NestedMICA distances	MEME distances	NestedMICA MCCs	MEME MCCs
m4 + m7	40	0.23, 0.45	11.73, 0.53	0.74, 0.93	0.02, 0.92
	20	0.54, 0.62	11.73, 0.56	0.71, 0.93	0.02, 0.94
m4 + m10	40	0.44, 0.75	11.73, 0.46	0.81, 0.95	0.02, 0.96
	20	0.34, 0.73	11.73, 0.75	0.75, 0.96	0.02, 0.96
m7 + m10	40	0.47, 1.11	0.38, 0.45	0.95, 0.96	0.94, 0.95
	20	0.71, 0.75	0.70, 0.62	0.93, 0.95	0.92, 0.95
m4 + m7 + m10	40	0.42, 1.01, 1.00	11.73, 0.44, 0.42	0.75, 0.95, 0.97	0.02, 0.93, 0.96
	20	0.64, 0.54, 0.57	11.73, 0.76, 0.82	0.71, 0.95, 0.97	0.02, 0.93, 0.95

### Performance vs. protein length

Having performed the motif spiking tests, in order to evaluate the two programs in a more natural situation, we observed the effects of varying sequence length on motif finding in multiple protein sets expected to contain C-terminal motifs. To this end, we used 198 non-redundant ER proteins (see Methods), a high proportion of which would be expected to contain the C-terminal ER retention signal mentioned above. We created three datasets containing sequence chunks of 60, 80 and 100 amino acid letters, respectively, taken from the C-terminal regions of these ER proteins.

**Figure 4:**
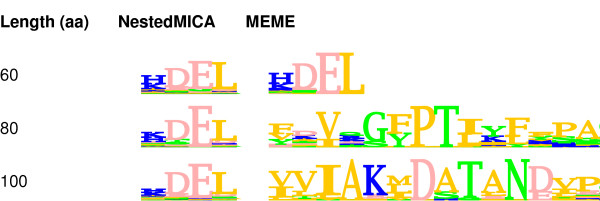
**Motif recovery performance against sequence length**. Shows recovered motifs using NestedMICA and MEME. "Length" refers to how many amino acid letters from the right-most (C-terminal) part of sequences were used in each dataset created. The 4 amino acid long ER retention signal was recovered successfully by NestedMICA while MEME reported motifs of the maximum allowed length (given by the user) when the sequences were longer than 80 residues.

Figure 4 depicts the motifs recovered from these three datasets by both programs. While MEME could not find the [KH]DEL motif at the tested sequence lengths of 80 and 100 amino acids, NestedMICA performed well, even when 100 amino acid long chunks were used. Both programs were run with default protein parameters with a target motif length set to between 3 and 15 amino acids.

To investigate whether NestedMICA would still find the motif when there are more than 100 residues per sequence, we tested it using 120 residue long C-terminal regions. The ER retention motif was found only when NestedMICA was asked to find two motifs. Investigating the other reported motif, we found that it was a thioredoxin family active site motif (Prosite id: PDOC00172) that is usually found in ER proteins. MEME was also tested when forced to find two motifs from the dataset containing the 80 amino acid long sequences. However, in addition to the motifs shown in Figure 4, it reported a 15 residue long motif which we could not locate in domain databases. Scanning this motif against the sequences, we noticed that it exists in 8 of the 198 proteins in the dataset.

### "Null test" and significance of motifs

For motif discovery assessment purposes, performing spiking motifs into a dataset of sequences that already contained strong motifs would be undesirable, as the method in question might report some of these intrinsic motifs instead of the artificially implanted ones. On the other hand, evaluating a motif discovery tool using a dataset of randomly generated sequences would be unfair, too, as this would be relatively easy for the program to recover a test motif.

Given that even sequences having a low sequence identity can in theory share some common sequential features, it is important to ensure that an unbiased set of sequences is used in the tests. For this reason we used non-homologous cytoplasmic sequences from the TargetP subcellular localisation dataset for these tests. This dataset had been already filtered by the TargetP developers using a homology reduction algorithm that ensures no homologous sequences exist in the set [19], before we filtered it again to further reduce the maximum sequence identity between any of the sequences.

We ran both NestedMICA and MEME on this dataset, before it was spiked by any test motifs, using different minimum target motif lengths for each program tested. This "null test" was performed, to confirm that the dataset we used in performing motif spiking tests is a reasonably suitable one. This "negative control" test also gives an idea about how well the trained background model represented the sequences.

For this purpose, NestedMICA was run with the default parameters optimised for protein sequences (for more details on the parameters, please see the program manual). In this test, the minimum target length was initially set to 2, then 3, and finally 4, while the maximum length was always kept as 15, as in the motif spiking tests. Motifs generated by NestedMICA from these runs were weak and short ones, having average information bit scores per position not exceeding 1.2 out of the possible 4.32 bits per position, which corresponds roughly to less than one third of the maximum height in a sequence logo (data not shown). This indicates that NestedMICA does not generally report false positive motifs, and that the chosen background model parameters are good enough to represent the test set. As we have seen above, NestedMICA is sensitive enough to report even scarce motifs of length 3 when present in only 10% of the sequences, as the examples in Figure 2 and Additional files 1, 2 indicate. Therefore, the fact that NestedMICA only reports weak "null test" motifs increases our confidence that the cytoplasmic sequence set that we use to assess motif discovery performance is not likely to contain significant motifs that a motif finder would prefer to report over any of our spiked motifs.

MEME, on the other hand, generally tended to report high-information containing motifs of the maximum allowed length, corresponding to about 46 bits in total, and above 3 bits per residue position. To minimise any remaining common patterns in the sequence set, we further reduced the maximum sequence identity within the set to 30%. Furthermore, all sequence regions matching a Prosite pattern were removed, based on hits reported by an annotated motif search tool PPSearch [20]. However, even with this extra filtered dataset, MEME still reported strong and long motifs similar to the 15 amino acid long ones in Figure 2.

When the user-specified number of target motifs exceeds the number of actual motifs, NestedMICA has been observed to generate motifs that look like the "null motif" of that particular dataset (data not shown). Similarly, MEME produced the same type of long motifs it found in the null tests when it failed to find an inserted motif in the spiking tests.

## Conclusion

We have added support for protein motif discovery in NestedMICA. It reports protein motifs in the form of PWMs. It has been optimised for better protein motif discovery under stringent conditions, and automatic motif length adjustment. In summary, our performance assessment tests show that NestedMICA performs very well when finding single and multiple motifs even at low motif abundance rates and different motif lengths, thus proving itself to be a robust and sensitive protein motif finder. Judging from the calculated sensitivity, specificity and MCC values, there was no clear difference regarding the quality of motifs correctly recovered by NestedMICA or MEME. However, when it comes to the number of correctly recovered motifs, NestedMICA significantly outperformed MEME in our protein motif finding tasks including finding low abundant motifs, finding short motifs, and finally discovering motifs from amino acid sequences of different lengths.

In addition to assessing its ability in finding true positive motifs, as shown in the results section, by running it on a non-redundant dataset where no test motif was inserted, we have shown that NestedMICA does not tend to report high-information content motifs when there is no meaningful motif contained in the dataset, i.e. that it tends not to report strong false negatives.

Considering that some protein signals such as subcellular localisation motifs could be as short as 3 amino acids, this new protein motif finder is a promising tool in functional sequence annotation.

## Methods

### The structure of NestedMICA

NestedMICA is a probabilistic motif inference method based on a generative sequence model. The model has three sets of parameters: firstly, a background model which represents all the non-motif parts of the input sequences; second, a set of position-weight matrices which represent the motifs themselves; finally, a binary matrix (the occupancy matrix) whose elements specify whether a given motif should be considered when modeling a given input sequence. The background model is built in advance and held constant during motif inference, while the motifs and occupancy matrix are updated to fit the supplied data. NestedMICA uses the Nested Sampling strategy [15] to update both these sets of parameters.

The implementation of NestedMICA's nminfer program can be split into two major parts: code that calculates the likelihood of some sequences under the generative model, and code which implements the Nested Sampling process. The Nested Sampling code makes few assumptions about the internal structure of the model (and could potentially be used to perform inference of quite different models), so we consider these two components separately.

### The NestedMICA sequence model

Interesting motif regions and the remaining uninteresting parts of sequences can be represented as Hidden Markov Models (HMMs). This kind of model can be referred to as a sequence mixture model (SMM), as they contain states representing motifs as well as some prior models. An example to SMMs is the zero-or-one occurrences per sequence (ZOOPS) model which is the default strategy in most motif finders based on expectation maximisation [21], or Gibbs sampling [22], a typical example of which can be the MEME [13] motif discovery program.

NestedMICA relaxes the constraints of this model slightly by allowing a given motif to appear multiple times in the same input sequence. To calculate the likelihood of a given sequence, NestedMICA first consults to appropriate row of the occupancy matrix to determine a (possibly empty) subset, *M*, of the complete motif set which applies to this sequence. In the case where *M *is empty, the likelihood of the sequence is simply its likelihood under the background model (see below). When *M *is non-empty, NestedMICA sums over all possible configurations of motif occurrences along the sequence, filling in any gaps using the background model. This is performed using a dynamic programming recursion which gives the likelihood, *L*_*n *_of all paths up to a given point in the input sequence, *n *as:

(1) $$L_{n}=(1-t)B_{n-1}L_{n-1}+\frac{t}{\left|M\right|}\sum_{m\in M}m(S_{n-\left|m\right|+1}^{n-1})L_{n-\left|m\right|}$$

where |*M*| is the number of motifs selected by the occupancy matrix, |*m*| is the length of weight matrix *m*, *B*_*n *_is the probability that the sequence symbol at position *n *was emitted by the background model, *m*($S_{i}^{j}$) is the probability that the sequence from *i *to *j *was emitted by the weight matrix *m*, and *t *is a transition probability specifying the estimated density of motifs in the sequence.

We initialise *L*_0 _= 1 then apply the above formula recursively along the length of the input sequence until the final position is reached, giving a likelihood for the complete sequence.

In principle, any background model could be used with this formulation. In practise, we choose to use a mosaic background [14] which admits the possibility of several different classes of background sequence, each of which is modeled using a low-order Markov chain (*i.e*. within a given class, the probability of observing a particular symbol at position *n *depends on the symbols observed at a fixed number of previous positions). The mosaic model is implemented as a fully connected HMM (transitions are allowed between any pair of classes).

To calculate *B*_*n*_, NestedMICA first applies the standard posterior decoding algorithm [23] to find *P*_*hn*_, the posterior probability that the symbol at position *n *in the input sequence was generated by state *h *of the background model *H*. We then calculate *B*_*n *_as:

(2) $$B_{n}=\sum_{h\in H}P_{hn}h(S_{n})$$

(*i.e*. summing over any remaining uncertainty in which background class is used at *n*). Note that when the Markov chain order, *o *is greater than zero, the probability of observing a given symbol, *h*(*S*_*n*_), depends on *o *previous symbols in the sequence. This means that is not possible to exactly calculate *B*_*n *_where *n *≤ *o*. We choose to ignore the first *o *symbols in the input sequence (except for background calculation purposes) in order to avoid any edge effects.

### Inference by Nested Sampling

Inferring optimal parameters for probabilistic models is a difficult task, particularly when the number of model parameters becomes large. NestedMICA performs inference using Nested Sampling [15], a robust Bayesian sampling method for model selection and parameter optimisation. Nested Sampling is a Monte Carlo inference strategy which can find globally good solutions to high-dimensional problems. Classical Monte Carlo methods work by moving a single state (*i.e*. set of parameters) around the problem's parameter space, accepting or rejecting proposed moves depending on whether they increase or decrease the likelihood of the observed data. Nested Sampling is always applied to an ensemble of *e *different states, where the value of *e *is typically a few hundred. The process starts with an ensemble of states sampled uniformly from the prior.

Having sampled the states, they are then sorted in order of likelihood, and the least likely state is removed from the ensemble. To maintain the ensemble size, a new state is sampled, subject to the constraint that the new state must have a likelihood greater than that of the state it is replacing. Repeating this process many times means that nested samplers progressively move towards a small subset of the state space which contains high-likelihood states. This is somewhat analogous to simulated annealing methods where a temperature parameter is reduced to bring the model progressively closer to the posterior distribution, but nested sampling avoids the need to explicitly cool the model: progress towards high-likelihood states occurs automatically.

For each step of Nested Sampling, a certain fraction of state space is removed from further consideration (since it contains states with likelihoods lower than the threshold). Over many steps, the fraction of prior mass that is removed from consideration at step *t *will tend towards

(3) $$W_{t}=\frac{1}{e}{\left(\frac{e}{e+1}\right)}^{t}$$

where *e *is the ensemble size. Since all the states which have been removed from consideration will have a likelihood of approximately *L*_*t*_, the likelihood of the state which was removed at step *t*, the Bayesian evidence for the model, *Z*, can be estimated as:

(4) $$Z=\sum_{t=1}^{\infty }W_{t}L_{t}$$

Clearly, it is possibly to progressively accumulate an estimate of *Z *during the Nested Sampling process. The final estimate of *Z *can be used for model comparison purposes (for example, finding optimal parameters for the NestedMICA sequence model). NestedMICA also uses *Z*_*t*_, the online *Z *estimate up to step *t *to decide when to terminate the Nested Sampling process. Specifically, we terminate when:

(5) $$\frac{1}{Z_{t}}L_{t}{\left(\frac{e}{e+1}\right)}^{t}<0.01$$

*i.e*. the likely increase of *Z *in future iterations is small compared to the current value. Formally, this may lead to premature termination if *L *increases dramatically late in the training process, but in practise we find that this simple criterion is effective for motif discovery.

### Implementation of NestedMICA

The NestedMICA nminfer program is based around a fairly general implementation of the Nested Sampling strategy, which can be applied to any probabilistic model. This code takes three inputs: a data set (*i.e*. a set of sequences), some code to calculate the likelihood of the dataset given a model state (*i.e*. an implementation of the likelihood function given above), plus a set of "sampling" operations which perturb a state and can be used to move around state space.

Each state consists of two sets of parameters: a set of motif weight matrices, and an occupancy matrix specifying where the motifs appear in the input sequence set. Most of NestedMICA's sampling moves are applied to one randomly selected weight matrix (WM):

• making a small perturbation to one column of a weight matrix, by slightly increasing or decreasing one of the weights, then renormalising so they still sum to 1.

• replacing a WM column with a new one, sampled from the prior.

• removing a column in one end of a WM while adding another one to the other end.

• adjusting motif length, by adding or removing a column from either end.

In addition, it is necessary to resample the occupancy matrix. In principle, a straightforward and valid sampling move would be to simply flip the state of one randomly-selected element in the occupancy matrix. In practise, NestedMICA tests multiple occupancy matrix moves at the same time, since this improves performance when running on multi-processor systems.

Finally, it is necessary to place a prior over the state space. NestedMICA uses a simple non-informative prior for the Weight Matrix motif models: a uniform prior over weight-matrix space with a constraint that extremely low weights are forbidden. The lower limit is specified by the -minClip parameter and is typically 10^-7 ^for amino acid, and of the order of 10^-3 ^for dna input. We also place a non-informative prior on the occupancy matrix, although if there is some prior knowledge about the frequency of the target motif in the dataset, this can be specified using the -expectedUsageFraction option.

### Adding protein support to NestedMICA

We made several changes to NestedMICA in order to support protein motif discovery. Firstly, we added support for loading and analysing protein sequences (enabled with the -alphabet PROTEIN switch). The inference strategy remains identical to that previously described [14]. However, the dimensionality of the protein motif discovery problem is much higher than in nucleic acids: a DNA motif model has three free parameters per position, while a protein motif has 19. To compensate for this difference, we found that a rather larger ensemble of models in the Nested Sampling process was required than for DNA. Having found an optimal ensemble size by performing a systematic parameter sweep test, we altered this to be the default ensemble size when running the program in protein mode.

Another important difference between the protein-capable version and the previous version of NestedMICA is the way distribution probability initialisation is performed in setting up the amino acid probability distributions for each background mosaic class. Starting off with flat probability distributions in all the mosaic classes of a given background as in the dna case was not ideal for protein sequences, as we observed a minimal learning rate with these equal initial states. Instead, a semi random, semi actual input-based initialisation was preferred: The distributions were initialised such that they directly reflect the amino acid distributions of the actual input data, except, these numbers were slightly changed randomly by a certain margin for the training to learn and converge faster.

Since the initial publication of NestedMICA [14], an important extra feature was added of automatically optimising a motif's length within a user-specified motif length range. NestedMICA treats the motif length as another free parameter of the motif model, and optimises it using the same Nested Sampling strategy as for all the other parameters. Another change in the new version is that, if no background model is provided by the user, NestedMICA uses a basic, zero-order background model which is trained on the fly from the user supplied input sequences.

Further information regarding the parameters used in motif finding can be found in the user manual at the NestedMICA web site [24].

### Background model training

Probabilistic motif finding tools usually employ background models to represent sequence regions where ideally no motif of interest exists. In most cases, however, these programs use a homogenous background model, assuming that all non-motif portions of the sequence can be represented using a single amino acid frequency distribution. In reality, protein sequences are generally composed of different functional domains which can be chemically biased towards certain compositional forms. In addition, protein sequences are very likely to carry different sequence signals responsible for various molecule-recognition and binding related tasks. NestedMICA uses non-homogenous ("mosaic") background models which sub-divide the background sequences into several classes. Each class is modelled as a Markov chain. The order of the chain (i.e. the number of previous symbols on which the probability distribution for the next observed symbol is conditioned) can be set to an arbitrary value, but for protein sequence analysis we recommend only using zeroth or first-order background models, since higher order models will have an extremely high parameter count and will be hard, if not impossible, to parametrise effectively.

A built-in background likelihood estimation procedure in NestedMICA (called "nmevaluatebg") allows an optimal background model architecture to be found for a given set of sequences. A NestedMICA background model can be of any order Markov chain and, consist of an arbitrary number of mosaic classes. As a good representative sequence set, we used the pTarget protein subcellular localisation dataset [25] for background model parameter optimisation (Figure 1). This is mainly because it includes different types of proteins from different subcellular localisations, eliminating the chance of some strong domain and localisation signals to overarch the background model training and evaluation. Furthermore, we reduced the sequence identity of the set from 95% down to a maximum of 40% by using the CD-HIT [26] clustering software to have a total of 7437 eukaryotic proteins, which had an average sequence length of 522. For evaluation purposes, 6000 of these were used to train several different background models with different parameters, while the remaining sequences were used to test how well a certain background model represented them. As Figure 1 shows, using order-1 probabilities, where the compositional probability of a certain residue depends only on a single adjacent residue, performs better than a zero-order model. Moreover, likelihood for the test sequences increased monotonically with the number of mosaic classes. Training a multi-class higher-order background requires sufficient sequence data in order to prevent a possible over-fitting of the background. For example, using a first order, 6-classes model corresponds to having a total of 2400 different amino acid distributions.

### Program output and sequence logos

NestedMICA reports discovered motifs as PWMs which can be viewed as sequence logos by an accompanying motif-viewer tool. In a single NestedMICA protein motif logo, each column has a maximum information content of 4.32 bits (*log*_2_20), and amino acid letters are coloured according to their general physical and chemical properties, as depicted in Figure 2.

As opposed to majority of motif finders, NestedMICA does not report any significance measures such as E-values, or entropy scores, as these values could be quite unreliable. All these scores are calculated based on the idea that a motif finder has picked up a real motif, which obviously cannot always be true. The recent publication by [27], discusses in detail why using such scores could lead to undesirable results.

### Testing NestedMICA's performance

In order to get a better understanding of NestedMICA's protein motif finding capabilities and limits, a number of motif spiking experiments were performed using synthetic and biological motifs, similar to the approach previously used by [14]. In a motif spiking test, a number of short amino acid sequences are generated according to the weight matrix distribution probabilities of a given motif. These motif-resembling short peptides are then inserted at random positions into a set of sequences. The program under test is then applied to the set of sequences to predict a set of motifs. Finally, the predicted candidate motif set is compared with the original test set to assess the performance of the program in recovering the spiked motifs. MEME PWMs were converted into NestedMICA sequence logos for easier comparison.

To evaluate how similar a reported motif is to the original one, we used cartesian motif-motif distances. The cartesian motif distance metric is the sum of individual cartesian distances calculated for each motif position, between corresponding pairs of the 20 amino acid probabilities from both motifs. For a motif to be considered as recovered with a reasonable precision, we used an empirically set threshold for the maximum allowed cartesian motif distance normalised for the original motif length. Motifs showing an average deviation per position of more than 0.3 of cartesian motif distance were considered as false discoveries.

For each reported motif, in addition to reporting its cartesian motif distance to the original test motif, we calculated its sensitivity (SN) and specificity (SP) values:

(6) $$SN=\frac{TP}{TP+FN}$$

(7) $$SP=\frac{TP}{TP+FP}$$

Matthew's Correlation Coefficient (MCC) [18] values were calculated, too, to show a PWM's scanning power as in [28]:

(8) $$MCC=\frac{TP\;TN-FN\;FP}{\sqrt{\left(TN+FN)(TP+FN)(TN+FP)(TP+FP\right)}}$$

where TP, FP, FN, TN stand for true positives, false positives, false negatives and true negatives, respectively.

One advantage of using MCC in a PWM evaluation is that for random motif predictions MCC tends to be around zero, while for a perfect scanning performance it will have a maximum value of 1. On the other hand, depending on the choice of a score threshold, even for an irrelevant or weak motif one can get a sensitivity of 1, for instance, while the corresponding specificity value could be as low as 0.5, if the number of sequences in both datasets are equal. In such cases, MCC will tend to be very low, reflecting the random prediction.

To calculate these measures of motif scanning performance, first, we spiked every sequence in the test dataset with a particular motif, then we scanned a reported motif both in the spiked and original datasets to see how many motif instances would be correctly or falsely predicted in both datasets. For each individual test case, we picked a threshold score that maximises the corresponding MCC value, after trying a range of different score thresholds systematically incremented in each iteration to compute sensitivity, specificity and MCC values. We calculated these values not only for motifs reported by the programs we assessed, but also for the original test motifs. We did this because values measuring the scanning performances of recovered motifs should be considered relative to those of the original motif. A more objective and absolute metrics of motif recovery is the cartesian motif distance, which is the sum of probability differences in corresponding columns of any two compared motifs. For example, a test motif which contains only a small number of strongly conserved residues cannot be expected to have a good scanning performance in identifying all spiked motifs, because the motif tolerates too much sequence variation. Therefore judging the performance of a motif discovery tool based on only such sensitivity/specificity measures is inadequate, since a motif tool should find a weak motif from a set of spiked data, if the original motif is a weak one, too. The sensitivity/specificity of this type of less conserved motifs would be relatively low, and not reflect or reward a program's ability to have discovered such a difficult motif. Therefore, we report MCC of the original test motifs primarily as a measure indicating how difficult a motif is to recover by a motif discovery program, and we report cartesian motif distances with the purpose of indicating how good the program is in that task. For instance, even an MCC value of 0.6 would still be good for a motif found by a program, if the corresponding real test motif did not have a much better MCC.

To generate test motifs for the program's assessment, we used conserved blocks of several ClustalW multiple alignments of sufficiently large number of Swiss-Prot [29] proteins which all feature an arbitrarily chosen Prosite [3], or PFAM domain entries. Segments from these domains' alignments were converted into PWMs to obtain 3 sets of 7 test motifs of varying lengths between 3 and 9. The 21 test motifs used in the evaluations are available for download at the NestedMICA home page.

As a dataset to carry out the spiking tests on, we used 438 whole-length cytoplasmic protein sequences obtained from the redundancy-reduced non-plants version of the TargetP [19] subcellular localisation dataset. Having an average sequence length of 582, this dataset does not include any homologous proteins, after a filtering process by an algorithm mentioned in [19]. Both NestedMICA and MEME were run with the default options. Note that, NestedMICA's default parameters differ from those used in DNA motif finding. Both NestedMICA and MEME require a target motif length interval, and no matter what the actual spiked motif's length was, for all of our spiking tests this was set to be between 3 and 15.

The background model used in the spiking tests was trained from the same cytoplasmic sequence dataset. The similar background likelihood analysis that was performed on another set (Figure 1) suggested that there would be no significant gain in likelihood when using a model with more than 4 mosaic classes for this particular small dataset. Therefore, a first order background model containing 4 mosaic classes was used in the tests.

Finally, for the evaluation of the program's assessment in subcellular localisation motif recovery, which was performed using sequences of different lengths, we used the ER dataset of a multi-class protein subcellular localisation predictor, MultiLoc [30]. This dataset contains 198 homology-reduced, eukaryotic ER proteins.

## Availability and requirements

• Project Name: NestedMICA

• Project home page: 

• Operating systems: Platform independent

• Programming languages: Java and C

• Other requirements: Biojava1.4, StAX-compliant XML parser (all included within the NestedMICA package), ANT 1.7.0  to compile the project

• License: LGPL

• Any restrictions to use by non-academics: None

## List of abbreviations

• ER: Endoplasmic Reticulum; • HMM: Hidden Markov Model; • MCC: Matthew's Correlation Coefficient; • PWM: Position Weight Matrix; • SP: Signal Peptide; • WM: Weight Matrix.

## Authors' contributions

TH and MD conceived this work, MD and TD modified the NestedMICA code, MD performed the tests and wrote the manuscript. All authors read and approved the final manuscript.

## Supplementary Material

Additional file 1 Motifs recovered by NestedMICA and MEME in the single-motif spiking tests, for motif set 2. This file contains a figure showing the second set of test motifs as recovered by the two compared programs, along with their cartesian distances to the original motifs and their MCC values. Click here for file

Additional file 2 Motifs recovered by NestedMICA and MEME in the single-motif spiking tests, for motif set 3. This file contains a figure showing the third set of test motifs as recovered by the two compared programs, along with their cartesian distances to the original motifs and their MCC values. Click here for file

Additional file 3 Sensitivity and specificity values for the motifs reported by NestedMICA and MEME. This file contains two tables showing sensitivity and specificity values of the compared programs in the single and multiple motif spiking tests. Click here for file
