# Supplementary material for: NestedMICA as an ab initio protein motif discovery tool
Source: BMC Bioinformatics. 2008 Jan 14;9:19. doi: 10.1186/1471-2105-9-19 (PMC2267705; doi:10.1186/1471-2105-9-19)
Supplement: Additional file 1 — Motifs recovered by NestedMICA and MEME in the single-motif spiking tests, for motif set 2. This file contains a figure showing the second set of test motifs as recovered by the two compared programs, along with their cartesian distances to the original motifs and their MCC values. [file 1471-2105-9-19-S1.pdf]

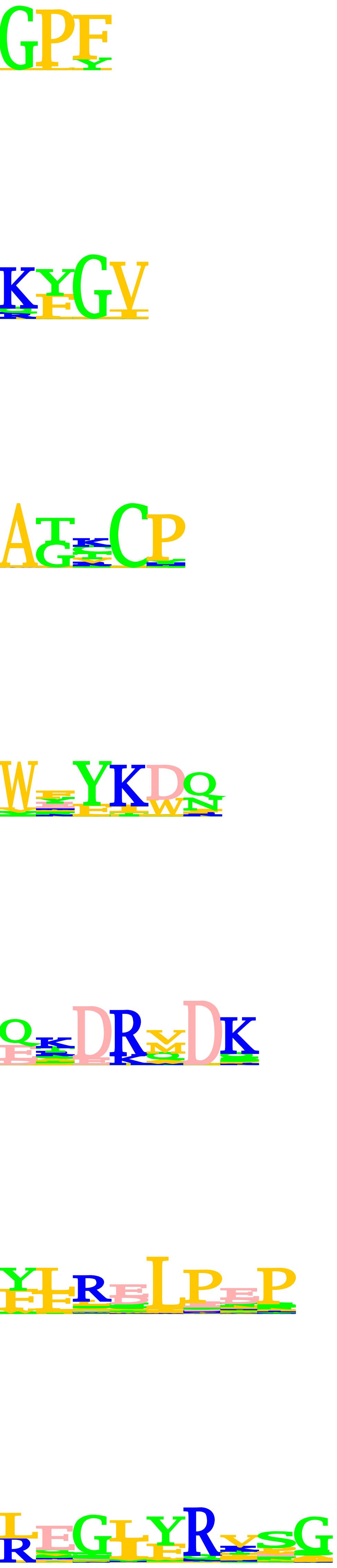

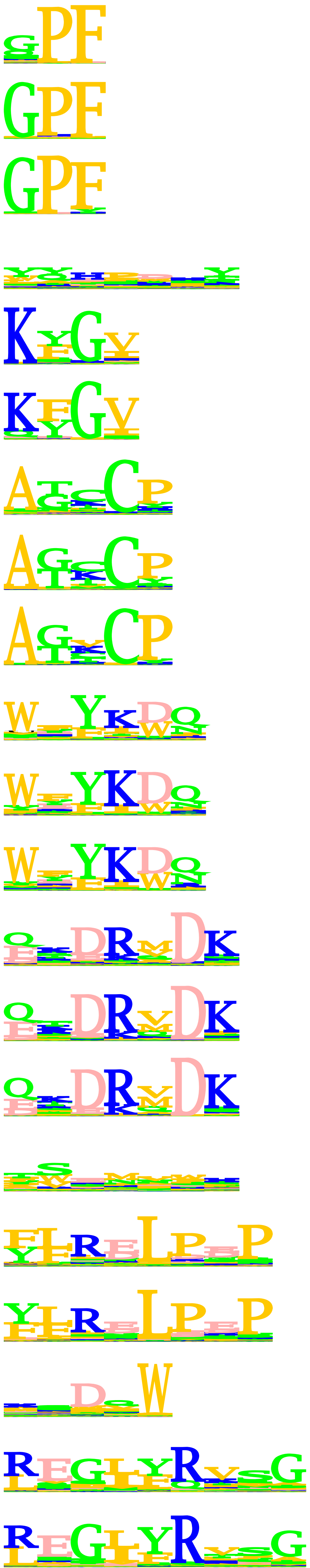

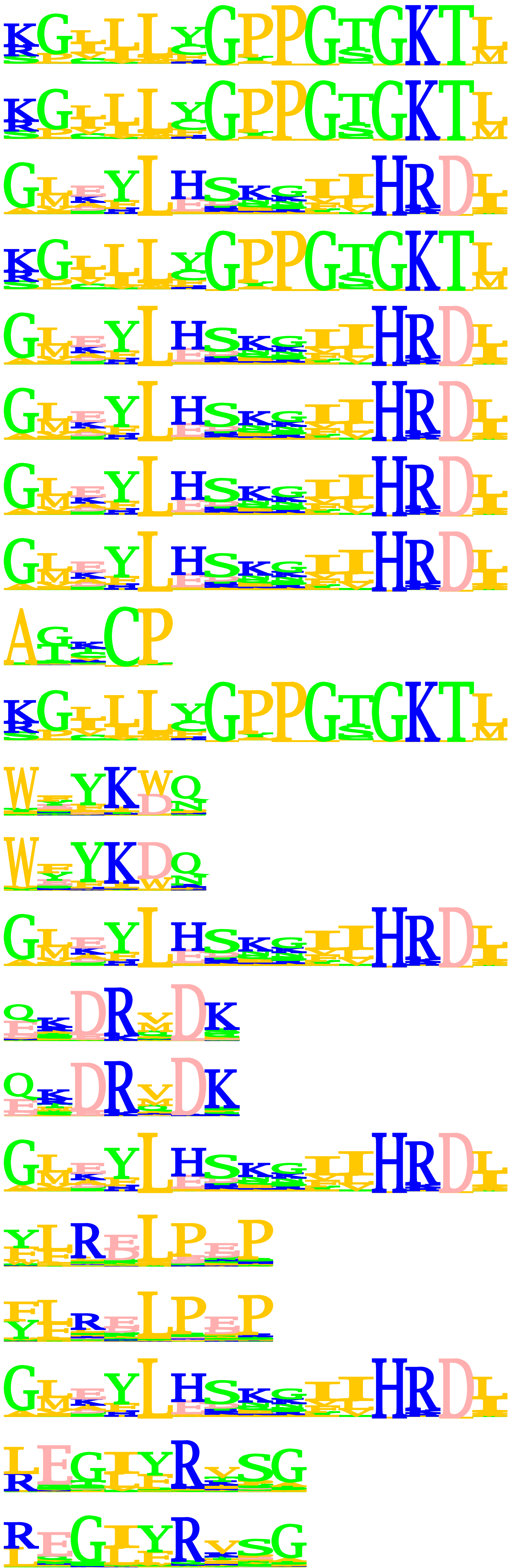

Original motif	Abundance	MCC for original	NestedMICA	Distance & MCC for NestedMICA		MEME	Distance & MCC for MEME	
	10	0.850		0.84	0.726		10.75	0.040
	20			0.35	0.726		10.75	0.040
	30			0.26	0.850		10.67	0.015
	10	0.822		4.50	0.018		11.73	0.029
	20			0.69	0.693		11.16	0.010
	30			0.32	0.693		11.16	0.010
	10	0.921		0.76	0.931		12.05	0.015
	20			0.50	0.936		12.05	0.015
	30			0.29	0.884		0.29	0.874
	10	0.884		0.81	0.911		12.77	0.014
	20			0.51	0.858		0.72	0.878
	30			0.47	0.886		0.57	0.853
	10	0.943		1.03	0.939		11.31	0.013
	20			0.64	0.939		0.57	0.930
	30			0.45	0.948		0.59	0.920
	10	0.830		5.45	0.012		11.17	0.045
	20			0.71	0.810		0.67	0.806
	30			0.61	0.812		0.61	0.805
	10	0.941		6.85	0.016		11.76	0.019
	20			0.99	0.927		1.05	0.909
	30			0.67	0.932		0.71	0.935
