# Supplementary material for: NestedMICA as an ab initio protein motif discovery tool
Source: BMC Bioinformatics. 2008 Jan 14;9:19. doi: 10.1186/1471-2105-9-19 (PMC2267705; doi:10.1186/1471-2105-9-19)
Supplement: Additional file 2 — Motifs recovered by NestedMICA and MEME in the single-motif spiking tests, for motif set 3. This file contains a figure showing the third set of test motifs as recovered by the two compared programs, along with their cartesian distances to the original motifs and their MCC values. [file 1471-2105-9-19-S2.pdf]

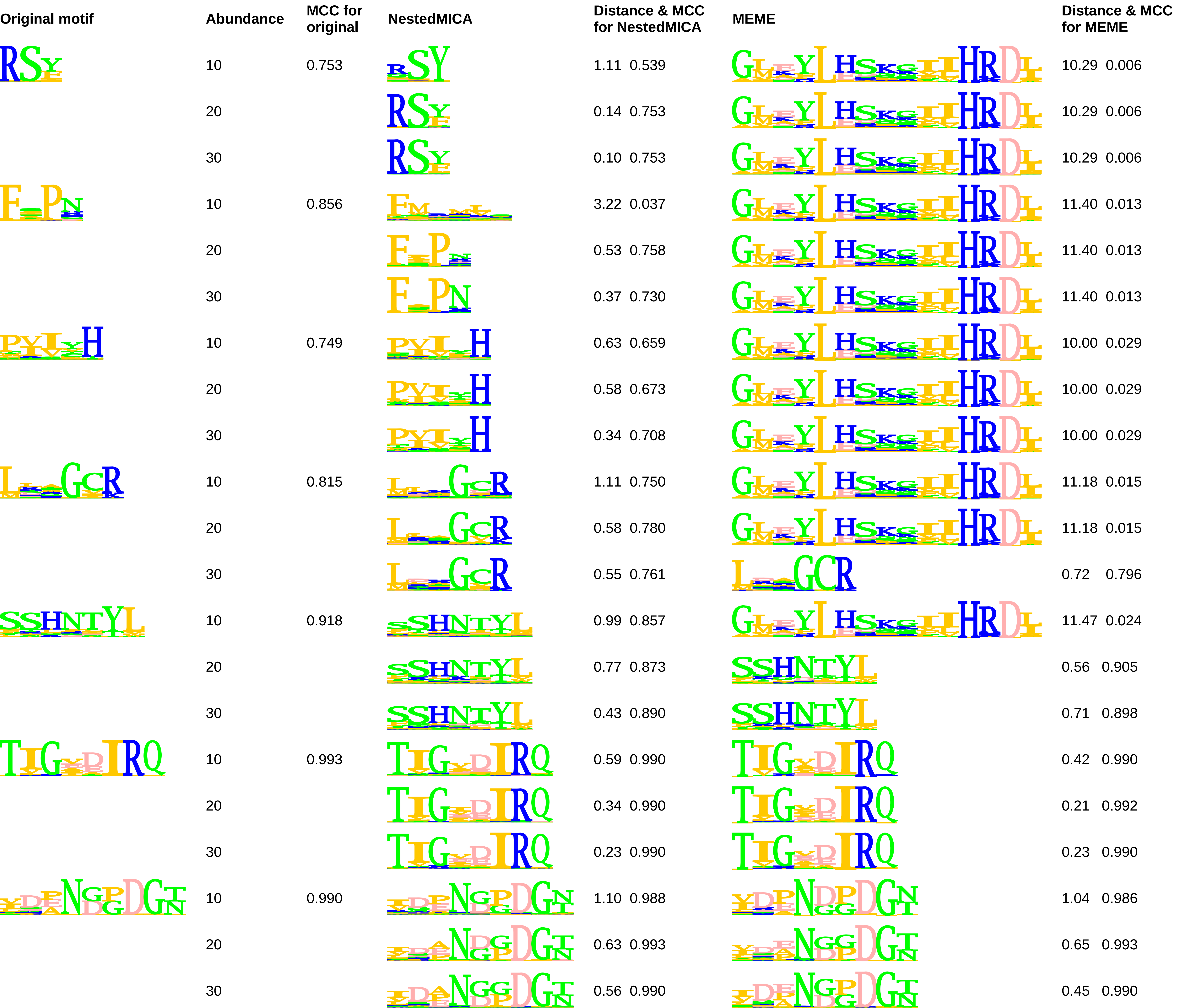

Original motif	Abundance	MCC for original	NestedMICA	Distance & MCC for NestedMICA	MEME	Distance & MCC for MEME
	10	0.753		1.11 0.539		10.29 0.006
	20			0.14 0.753		10.29 0.006
	30			0.10 0.753		10.29 0.006
	10	0.856		3.22 0.037		11.40 0.013
	20			0.53 0.758		11.40 0.013
	30			0.37 0.730		11.40 0.013
	10	0.749		0.63 0.659		10.00 0.029
	20			0.58 0.673		10.00 0.029
	30			0.34 0.708		10.00 0.029
	10	0.815		1.11 0.750		11.18 0.015
	20			0.58 0.780		11.18 0.015
	30			0.55 0.761		0.72 0.796
	10	0.918		0.99 0.857		11.47 0.024
	20			0.77 0.873		0.56 0.905
	30			0.43 0.890		0.71 0.898
	10	0.993		0.59 0.990		0.42 0.990
	20			0.34 0.990		0.21 0.992
	30			0.23 0.990		0.23 0.990
	10	0.990		1.10 0.988		1.04 0.986
	20			0.63 0.993		0.65 0.993
	30			0.56 0.990		0.45 0.990
