# Supplementary material for: NestedMICA as an ab initio protein motif discovery tool
Source: BMC Bioinformatics. 2008 Jan 14;9:19. doi: 10.1186/1471-2105-9-19 (PMC2267705; doi:10.1186/1471-2105-9-19)
Supplement: Additional file 3 — Sensitivity and specificity values for the motifs reported by NestedMICA and MEME. This file contains two tables showing sensitivity and specificity values of the compared programs in the single and multiple motif spiking tests. [file 1471-2105-9-19-S3.pdf]

		SET 1				SET2				SET3			
		NMICA		MEME		NMICA		MEME		NMICA		MEME	
Length	Abundance	SN	SP	SN	SP	SN	SP	SN	SP	SN	SP	SN	SP
3	10	0.988	0.855	0.995	0.501	0.770	0.933	0.197	0.506	0.559	0.903	0.197	0.506
	20	0.988	0.855	0.995	0.501	0.770	0.933	0.197	0.506	0.875	0.877	0.197	0.506
	30	0.988	0.855	0.995	0.501	0.950	0.904	0.197	0.506	0.875	0.877	0.197	0.506
4	10	0.811	0.545	0.197	0.506	0.930	0.503	0.993	0.501	0.921	0.506	0.993	0.501
	20	0.995	0.501	0.197	0.506	0.664	0.986	0.993	0.501	0.839	0.909	0.993	0.501
	30	0.847	0.728	0.197	0.506	0.842	0.850	0.993	0.501	0.775	0.934	0.993	0.501
5	10	0.487	0.914	0.592	0.507	0.978	0.953	0.854	0.506	0.731	0.897	0.854	0.506
	20	0.753	0.921	0.592	0.507	0.954	0.980	0.854	0.506	0.782	0.874	0.854	0.506
	30	0.782	0.921	0.592	0.507	0.974	0.914	0.854	0.506	0.837	0.866	0.854	0.506
6	10	0.950	0.501	0.978	0.501	0.921	0.987	0.995	0.501	0.839	0.902	0.995	0.501
	20	0.849	0.808	0.978	0.501	0.866	0.984	0.995	0.501	0.863	0.911	0.995	0.501
	30	0.703	0.913	0.978	0.501	0.914	0.969	0.856	0.932	0.794	0.948	0.856	0.932
7	10	0.995	0.501	0.995	0.501	0.947	0.990	0.139	0.532	0.906	0.947	0.139	0.532
	20	0.890	0.923	0.995	0.501	0.942	0.995	0.926	0.977	0.882	0.984	0.926	0.977
	30	0.823	0.958	0.818	0.950	0.962	0.985	0.928	0.968	0.902	0.984	0.928	0.968
8	10	0.957	0.968	0.856	0.507	0.959	0.501	0.995	0.995	0.995	0.995	0.995	0.995
	20	0.959	0.976	0.959	0.976	0.873	0.931	0.995	0.998	0.993	0.998	0.995	0.998
	30	0.971	0.964	0.964	0.969	0.873	0.933	0.990	1.000	0.993	0.998	0.990	1.000
9	10	0.974	0.514	0.990	0.502	0.995	0.501	0.986	1.000	0.993	0.995	0.986	1.000
	20	0.835	0.938	0.995	0.501	0.940	0.985	0.995	0.998	0.995	0.998	0.995	0.998
	30	0.875	0.915	0.851	0.939	0.938	0.992	0.995	0.995	0.995	0.995	0.995	0.995

Supplementary Table 1: Sensitivity (SN) and specificity (SP) values for the motifs reported by both NestedMICA (NMICA) and MEME for the single-motif spiking tests. Length refers to number of residue positions in motifs. SN and SP values are given for three sets of motifs we used, for each of the tested motif abundance rate which are given as percentages.

Motifs	Abundance	NMICA		MEME	
		SN	SP	SN	SP
m4 + m7	40	0.892, 0.949	0.855, 0.980	0.942, 0.947	0.503, 0.975
	20	0.685, 0.947	0.986, 0.980	0.942, 0.959	0.503, 0.982
m4 + m10	40	0.973, 0.964	0.856, 0.985	0.942, 0.980	0.503, 0.985
	20	0.745, 0.978	0.974, 0.980	0.942, 0.988	0.503, 0.978
m7 + m10	40	0.968, 0.976	0.982, 0.987	0.954, 0.980	0.982, 0.976
	20	0.932, 0.971	0.994, 0.983	0.949, 0.978	0.975, 0.973
m4 + m7 + m10	40	0.978, 0.968, 0.976	0.798, 0.985, 0.990	0.942, 0.952, 0.978	0.503, 0.980, 0.985
	20	0.685, 0.964, 0.978	0.986, 0.980, 0.987	0.942, 0.954, 0.976	0.503, 0.975, 0.978

Supplementary Table 2: Sensitivity (SN) and Specificity (SP) values for the motifs reported by both programs in the multiple motif spiking tests. The test were performed either at the total abundance rate of 40% or 20%. For the “m4 + m7” line, for example, the SN and SP columns show the corresponding values for motif of length 4 and motif of length 7 (Figure 3), respectively.
